# Supplementary material for: Remote testing of vitamin D levels across the UK MS population—A case control study
Source: PLoS One. 2020 Dec 30;15(12):e0241459. doi: 10.1371/journal.pone.0241459 (PMC7773187; doi:10.1371/journal.pone.0241459)
Supplement: S1 Fig — (a) The distribution of the 1768 study participants who provided questionnaire data. (b) The distribution of the MS participants selected to receive biological sampling kits. (DOCX) [file pone.0241459.s001.docx]

**S1 Fig.** Distribution of study participants across the UK. a. The distribution of the 1768 participants in the Recruited cohort. b. The distribution of the 668 MS participants selected to receive biological sampling kits.


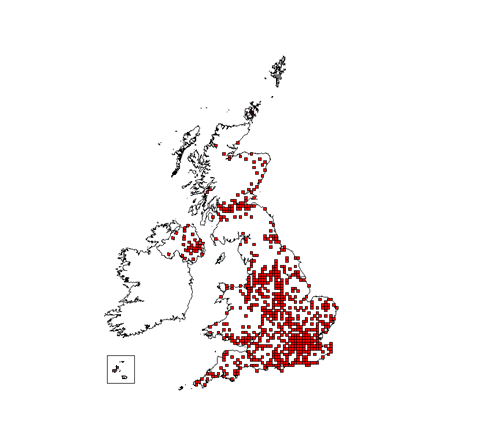


b.

a.


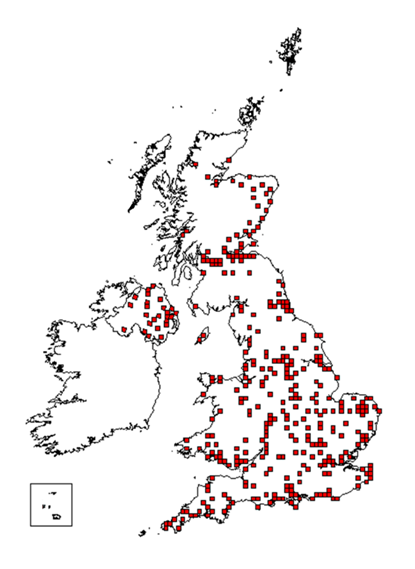


^ǂ^

^ǂ^

^ǂ Shetland islands, Orkney Islands, Outer Hebrides, Isle Man and Channel island participants not shown due to data protection^

^Statis map image created using ArcGIS. Map image is the intellectual property of Esri and is used herein under license. Used with permission as a static map in an academic publication. Map image copyright © 2020 Esri and its licensors. All rights reserved.^
