# Supplementary material for: Remote testing of vitamin D levels across the UK MS population—A case control study
Source: PLoS One. 2020 Dec 30;15(12):e0241459. doi: 10.1371/journal.pone.0241459 (PMC7773187; doi:10.1371/journal.pone.0241459)
Supplement: S1 Table — (DOCX) [file pone.0241459.s002.docx]

**S1 Table.** Comparison of demographic characteristics between those individuals who returned a sample pack and those who did not.

|  |  |  | |  |
| --- | --- | --- | --- | --- |
|  |  | **MS no sample pack return (n=233)^a^** | **MS sample pack return (n=388)** | **p-value** |
|  |  |  |  |  |
| **Sex** |  |  |  |  |
| Female n (%) |  | 155 (67) | 292 (75) | 0.019 |
| **Age** |  |  |  |  |
| Median years (IQR)^b^ |  | 53 (15) | 56 (14) | 0.003 |
| **MS type, n (%)** |  |  |  |  |
| RRMS |  | 101 (43) | 137 (35) |  |
| SPMS |  | 59 (25) | 120 (31) | 0.089 |
| PPMS |  | 58 (25) | 93 (24) |  |
| Other |  | 15 (7) | 38 (10) |  |
| **EDSS** |  |  |  |  |
| Median (IQR)^c^ |  | 6.5 (3) | 6.5 (3) | 1 |
| Low EDSS (<6): n (%) |  | 25 (40) | 41 (38) |  |
| High EDSS (≥6); n (%) |  | 38 (60) | 66 (62) | 0.86 |

^a^ No data for 47 non-participating as sampling packs were distributed via clinic and no data collected. ^b^Data missing for 4 non-participating and 5 participating MS. ^c^Data missing for 170 non-participating and 281 participating MS.
