# Supplementary material for: Remote testing of vitamin D levels across the UK MS population—A case control study
Source: PLoS One. 2020 Dec 30;15(12):e0241459. doi: 10.1371/journal.pone.0241459 (PMC7773187; doi:10.1371/journal.pone.0241459)
Supplement: S2 Table — (DOCX) [file pone.0241459.s003.docx]

**S2 Table.** Vitamin D supplementation behaviour and serum 25(OH)D levels in MS cases in the Recruited cohort.

|  | **Supplementing behaviour of MS (n=1768)** | | | |
| --- | --- | --- | --- | --- |
|  | **Taking supplements**  **n^a^ (%)** | **p-value** | **Dose, median IU/day**  **(IQR); n^b^** | **p-value** |
| **Disease status** |  |  |  |  |
| MS | 1234 (70) |  | 2000 (3000);1012 |  |
| **Sex** |  |  |  |  |
| female | 915 (69) | 0.14 | 2000(3000);750 | 0.32 |
| male | 319 (73) |  | 2000 (4000);262 |  |
| **MS type** |  |  |  |  |
| RRMS | 692 (71) | 0.58 | 2000 (3500);575 | 0.007 |
| SPMS | 314 (68) |  | 1600 (3200);253 |  |
| PPMS | 144 (71) |  | 2000 (4000);117 |  |
| **MS disability** |  |  |  |  |
| low EDSS (<6) | 177 (72) | 0.12 | 2000 (3000);150 | 0.31 |
| high EDSS (≥6) | 169 (65) |  | 1600 (3200);138 |  |

^a^data was missing for the following: sex of 2 participants, MS type of 132 participants, EDSS of 1262 participants; ^b^out of the total n that provided supplementation data this n had a dose available.
