# Supplementary material for: Remote testing of vitamin D levels across the UK MS population—A case control study
Source: PLoS One. 2020 Dec 30;15(12):e0241459. doi: 10.1371/journal.pone.0241459 (PMC7773187; doi:10.1371/journal.pone.0241459)
Supplement: S3 Table — (DOCX) [file pone.0241459.s004.docx]

**S3 Table.** Multivariable analysis of factors influencing vitamin D dose of participants in the Recruited cohort and Sample cohort.

|  | **Description** | **B-coefficient (95% CI)** | **p-value** |
| --- | --- | --- | --- |
| Recruited MS | Years since diagnosis | -0.012 (-0.020 to -0.004) | 0.003 |
|  | Age | -0.002 (-0.009 to 0.005) | 0.48 |
| Sample MS | Years since diagnosis | -0.019 (-0.034 to -0.004) | 0.012 |
|  | Age | -0.008 (-0.005 to 0.029) | 0.25 |

Log transformation was applied to the response variable.
