# Supplementary material for: Remote testing of vitamin D levels across the UK MS population—A case control study
Source: PLoS One. 2020 Dec 30;15(12):e0241459. doi: 10.1371/journal.pone.0241459 (PMC7773187; doi:10.1371/journal.pone.0241459)
Supplement: S4 Table — (DOCX) [file pone.0241459.s005.docx]

**S4 Table.** Lifestyle factors and behaviours known to influence serum vitamin D in Sample cohort.

|  | **MS (n=388)**  **n^a^ (%)** | **Control (n=305)**  **n^b^ (%)** | **p-value** |
| --- | --- | --- | --- |
| **Diet type** |  |  |  |
| meat eater | 348 (90) | 289 (96) |  |
| vegetarian or vegan | 40 (10) | 13 (4) | 0.003 |
| **Oily fish consumption** |  |  |  |
| never | 67 (17) | 43 (14) |  |
| rarely | 137 (36) | 103 (34) | 0.59 |
| weekly | 156 (40) | 133 (44) |  |
| >weekly | 28 (7) | 25 (8) |  |
| **Outdoor activities** |  |  |  |
| rarely | 171 (44) | 41 (14) | <0.001 |
| >30min at least 1x/week | 97 (25) | 108 (36) |  |
| >30min at least 3x/week | 120 (31) | 154 (50) |  |
| **Sunblock** |  |  |  |
| never | 121 (31) | 40 (13) | <0.001 |
| rarely | 232 (60) | 195 (66) |  |
| weekly | 19 (5) | 33 (11) |  |
| >3x weekly | 14 (4) | 29 (10) |  |
| **Sunblock – cosmetic** |  |  |  |
| never | 195 (50) | 124 (41) | 0.05 |
| rarely | 109 (28) | 90 (30) |  |
| weekly | 22 (6) | 17 (6) |  |
| >3x weekly | 61 (16) | 69 (23) |  |
| **Sunblock – cosmetic** | ***Females*** | ***Males*** |  |
| never | 180 (35) | 139 (81) | <0.001 |
| rarely | 170 (33) | 29 (17) |  |
| weekly | 39 (8) | 0 |  |
| >3x weekly | 127 (24) | 3 (2) |  |
| **Sunblock – cosmetic** | ***Female MS*** | ***Female Control*** |  |
| never | 111 (38) | 69 (31) | 0.09 |
| rarely | 98 (34) | 72 (32) |  |
| weekly | 22 (7) | 17 (7) |  |
| >3x weekly | 60 (21) | 1. 30) |  |

^a^data was missing from the following: sunblock 2 participants, sunblock -cosmetic 1 participant; ^b^data was missing from the following: diet type 3 participants, oily fish consumption 1 participant, outdoor activities 2 participants, sunblock 8 participants, sunblock- cosmetic 5 participants.
