# Supplementary material for: Remote testing of vitamin D levels across the UK MS population—A case control study
Source: PLoS One. 2020 Dec 30;15(12):e0241459. doi: 10.1371/journal.pone.0241459 (PMC7773187; doi:10.1371/journal.pone.0241459)
Supplement: S5 Table — (DOCX) [file pone.0241459.s006.docx]

**S5 Table.** Multivariable analysis of variables influencing vitamin D serum 25(OH)D levels.

|  | **Description** | **B-coefficient (95% CI)** | **p-value** |
| --- | --- | --- | --- |
| Solar contribution  in non-supplementing cohort | Latitude | -0.120 (-0.184 to -0.056) | <0.001 |
|  | Outdoor^a^ |  |  |
|  | >30min/week | 0.061 (-0.203 to 0.326) | 0.65 |
|  | >30min 3x/week | 0.404 (0.120 to 0.688) | 0.007 |
|  | Month blood taken | 0.008 (-0.057 to 0.073) | 0.81 |
|  | Sunblock^b^ |  |  |
|  | rarely | 0.070 (-0.172 to 0.311) | 0.57 |
|  | weekly | 0.155 (-0.404 to 0.715) | 0.58 |
|  | >3x weekly | -0.505 (-1.14 to 0.128) | 0.12 |
| Non-supplementing MS | Age at study | -0.028 (-0.047 to -0.009) | 0.02 |
|  | BMI | -0.015 (-0.049 to -0.020) | 0.42 |
|  | Latitude | -0.196 (-0.376 to -0.016) | 0.06 |
|  | Outdoor^a^ |  |  |
|  | >30min/week | -0.150 (-0.589 to 0.289) | 0.52 |
|  | >30min 3x/week | 0.564 (0.002 to 1.13) | 0.08 |
| Supplementing MS | Dose (IU/day) | 25.8 (16.2 to 35.4) | <0.001 |
|  | Age at study | 0.501 (-0.500 to 1.50) | 0.33 |
|  | BMI | -0.877 (-2.50 to 1.50) | 0.30 |
|  | Latitude | 2.09 (-2.31 to 6.49) | 0.36 |
|  | Outdoor^a^ |  |  |
|  | >30min/week | 17.6 (-15.9 to 51.1) | 0.31 |
|  | >30min 3x/week | 9.74 (-10.1 to 29.6) | 0.34 |

^a^reference for outdoor ‘rarely’; ^b^reference category for sunblock ‘never’.

Log transformation was applied to the response variable.
