# Supplementary material for: Remote testing of vitamin D levels across the UK MS population—A case control study
Source: PLoS One. 2020 Dec 30;15(12):e0241459. doi: 10.1371/journal.pone.0241459 (PMC7773187; doi:10.1371/journal.pone.0241459)
Supplement: S6 Table — (DOCX) [file pone.0241459.s007.docx]

**S6 Table.** Validation cohort taken from UK Biobank.

|  | **MS (n=1978)** | **Controls (n=7912)** | **p-value** |
| --- | --- | --- | --- |
| **Demographics** |  |  |  |
| Age at recruitment, median years (IQR) | 55 (12) | 55 (12) | 1 |
| BMI, median kg/m^2^ (IQR) | 26.2 (6.1) | 26.4 (5.8) | 0.05 |
| **Ethnicity, n (%)** |  |  |  |
| White | 1937 (98) | 7748 (98) | 1 |
| **Sex, n, (%)** |  |  |  |
| Female | 1430 (72) | 5720 (72) | 1 |
| **Vitamin D supplementation** |  |  |  |
| Vitamin D – yes | 272 (14) | 465 (6) | <0.001 |
| Multivitamin – yes | 531 (27) | 1894 (24) | <0.001 |
| Vitamin D and/or multivitamin – yes | 697 (35) | 2169 (27) | 0.008 |
| **Oily fish intake, n, (%)** |  |  |  |
| Never | 248 (13) | 917 (12) | 0.35 |
| < 1x a week | 633 (32) | 2606 (33) |  |
| 1x a week | 726 (37) | 2964 (38) |  |
| 2-4x a week | 340 (17) | 1310 (17) |  |
| 5-6x a week | 11 (0.6) | 57 (0.7) |  |
| 1x or more daily | 8 (0.4) | 16 (0.3) |  |
| **Time spent outdoors, hours/day (IQR)** |  |  |  |
| Summer | 3 (2) | 3 (3) | <0.001 |
| Winter | 1 (2) | 1 (1) | <0.001 |
| **Sun protection, n (%)** |  |  |  |
| Always | 420 (21) | 1834 (23) | <0.001 |
| Do not go out in sunshine | 20 (1) | 43 (0.5) |  |
| Most of the time | 703 (36) | 3056 (39) |  |
| Never rarely | 186 (9) | 592 (7.5) |  |
| Sometimes | 648 (33) | 2382 (30) |  |
| **Vitamin D status** |  |  |  |
| Non-supplementing serum 25(OH)D, nmol/L median (IQR) | 42 (31) | 47 (30) | <0.001 |
| Supplementing serum 25(OH)D, nmol/L median (IQR) | 57 (30) | 58 (27) | 0.56 |
| Total cohort Serum 25(OH)D, nmol/L median (IQR) | 44 (32) | 47 (30) | <0.001 |
